# Mechanisms of organotropism in breast cancer and predicting metastasis to distant organs using deep learning

**DOI:** 10.1007/s12672-025-02905-5

**Published:** 2025-06-11

**Authors:** Meizhu Xiao, Zhijin Fu, Yanjiao Li, Min Zhang, Denan Zhang, Lei Liu, Qing Jin, Xiujie Chen, Hongbo Xie

**Affiliations:** Department of Pharmacogenomics, College of Bioinformatics Science and Technology, Harbin Medical University, Harbin, 150086 People’s Republic of China

**Keywords:** Breast cancer, Metastasis, Organotropism, Deep neural networks

## Abstract

**Background:**

Metastasis, the spread of cancer cells from the primary tumor to distant organs, is the leading cause of mortality in cancer patients. This process often exhibits a preference for specific organs, a phenomenon known as tumor organotropism. This study focuses on the organotropism of breast cancer and analyzes its genomic alterations following metastasis to four organs (bone, brain, liver, and lung). The research aims to explore the intrinsic characteristics of primary breast cancer and the interactions between tumor cells and the tumor microenvironment (TME) within these target organs. Building upon this foundation, we developed a deep learning model to identify organ-specific metastatic genes, providing insights into the molecular mechanisms of metastasis.

**Methods:**

To investigate the mechanisms of organ-specific metastasis in breast cancer, we employed an integrative approach combining single-cell RNA sequencing, bulk RNA sequencing, ChIP-seq data, and deep learning techniques. Single-cell analysis provided detailed insights into cellular heterogeneity and microenvironment interactions at metastatic sites. Bulk RNA sequencing enabled the identification of gene expression patterns associated with metastatic propensity. A deep neural network (DNN) model was developed to analyze these complex datasets and identify key predictors of organ-specific metastasis.

**Results:**

Our integrative analysis revealed distinct gene expression profiles and cellular compositions in metastatic lesions across different organs. We have identified that, regardless of the target organ, breast cancer metastasis critically depends on specific biological signaling pathways, including the MAPK signaling pathway, metabolic pathways, the PI3K-Akt signaling pathway, and the positive regulation of cell adhesion. Single-cell sequencing highlighted unique interactions between tumor cells and the microenvironment, which varied significantly depending on the metastatic site. Fibroblasts play a critical role in facilitating the colonization of breast cancer cells in metastatic organs. The deep learning models effectively identified key molecular signatures and pathways associated with organ-specific metastasis, providing insights into the metastatic process.

**Conclusion:**

The study underscores the importance of the tumor microenvironment in influencing breast cancer metastasis to distant organs. We also established a comprehensive framework for understanding the mechanisms driving organotropism metastasis in breast cancer. Additionally, we identified key genes and signaling pathways associated with organ-specific metastasis, providing insights that may inform future studies on risk assessment and potential therapeutic targets for metastatic breast cancer.

**Supplementary Information:**

The online version contains supplementary material available at 10.1007/s12672-025-02905-5.

## Introduction

Tumor metastasis is the main characteristic of advanced cancer, the most destructive stage of cancer progression. This process involves the evolution of tumor cells in vivo and interactions between tumor cells and the tumor microenvironment (TME). Furthermore, clinical studies have shown that tumor metastasis is organ-specific, also known as organotropism, where different types of tumors tend to metastasize to specific organs. For example, the liver is the most common organ involved in distant metastasis in patients with colorectal cancer (CRC) [1]. Lung cancer is the most common cancer that metastasizes to the brain and represents the leading cause of brain metastases in men. However, in women, breast cancer is the predominant source of brain metastases [2]. Furthermore, different types and subtypes of cancer exhibit unique patterns of organ-specific metastasis [3–5]. Therefore, elucidating the mechanism of organotropism in tumor metastasis can aid in the discovery of new therapeutic targets and strategies and is also an important research direction in precision medicine.

Breast cancer is a significant health threat and is one of the most common malignancies affecting women worldwide. Metastasis of breast cancer is notably common and follows a pattern of organotropism, where cancer cells show a predilection for certain organs. The common sites for breast cancer metastases include the brain, bones, liver, and lungs. Bone metastasis accounts for approximately 70% of breast cancer metastasis cases, followed by lung metastasis. This organ-specific metastasis is influenced by a variety of factors [6–8]. Yao et al. reported that osterix promotes bone metastasis in breast cancer by increasing the expression of various genes involved in the metastatic process. These results indicate that osterix is a promising biomarker for targeting and managing bone metastasis in patients with breast cancer [9]. Additionally, TGF*β* initially suppresses tumors but later promotes breast cancer bone metastasis by enhancing epithelial–mesenchymal transition, angiogenesis, and immunosuppression [10]. Although brain, liver and lung metastases in patients with breast cancer are not as common as bone metastases are, these organs have specific mechanisms underlying metastasis. For example, Claudin-2 can promote liver metastasis in patients with breast cancer [11]. Moreover, HER2 promotes breast cancer metastasis by increasing cell proliferation and survival, particularly leading to brain metastases, owing to its specific affinity for the central nervous system [12]. Therefore, understanding the mechanisms underlying this organotropism is crucial for developing targeted therapies that can more effectively manage metastatic breast cancer and improve patient outcomes.

Deep learning has essential application value in oncology research. Deep learning automatically learns and extracts features by constructing and training deep neural networks, thereby achieving efficient processing and analysis of complex data. Therefore, the use of deep learning to mine large-scale clinical and biological data, discover tumor metastasis biomarkers, and predict tumor metastasis risk has potential applications. Albaradei et al. constructed an AutoEncoder framework to learn nonlinear relationships between genes, using gene expression profiles from any primary cancer sample to predict whether the patient’s sample is primary or metastatic on the basis of a deep learning architecture [13]. Additionally, Yao et al. developed a multimodal deep learning model that integrates histopathological images, clinical data, and gene expression data to predict recurrence and metastasis risk in breast cancer patients [14]. These deep learning-based studies not only provide new insights into cancer metastasis research but also demonstrate that deep learning is a powerful tool for addressing cancer organotropism.

In this study, we aimed to further explore the complex characteristics of breast cancer at the single-cell and bulk levels, particularly to identify the key genes that play decisive roles in the organ-specific metastasis of breast cancer. These genes are crucial not only for understanding the biological behavior of breast cancer but also for potentially becoming new targets for future treatments. We focused on four common metastatic organs (bone, brain, liver, and lung) in breast cancer patients. By carefully analyzing the differences in the tumor microenvironment within these organs, we aimed to elucidate the unique mechanisms of tumor growth and evolution at different metastatic sites. Specifically, we explored how the organ-specific tumor microenvironment influences breast cancer metastasis and the colonization of tumor cells in distinct organs.

We found that positive regulation of cell adhesion, the PI3K‒Akt signaling pathway, the MAPK signaling pathway and metabolic pathways plays important roles in the process of breast cancer metastasis to other organs. Moreover, in breast cancer metastasis, the role of specific gene overexpression varies across different organs. The tumor samples from breast cancer patients with bone metastasis exhibited overexpression of CD44, leading us to speculate that CD44 may facilitate the colonization of breast cancer cells in bone. Similarly, we observed that the overexpression of TCF21 might support the colonization of breast cancer in the lungs. Additionally, the overexpression of APOC3 appears to contribute to the colonization of breast cancer in the liver.

By establishing a specific assessment model for breast cancer metastasis, we can gain a deeper understanding of organotropism in tumor metastasis and identify key molecular factors that may contribute to metastatic progression. To achieve this goal, we utilized a deep learning technology, deep neural networks (DNN). Additionally, we used Shapley Additive ExPlanations (SHAP) to explain the model. SHAP is an interpretable machine learning approach based on cooperative game theory [15]. It assigns each feature in a model an importance value by computing its marginal contribution to the prediction across different feature coalitions. Unlike conventional feature importance methods, SHAP provides a theoretically robust and consistent framework for interpreting complex machine learning models, including DNNs. By analyzing SHAP values, we identified key genes that drive metastasis to specific organs and assessed their influence on the DNN model. This model integrates patient genomic data with information on metastatic target organs to achieve a more comprehensive and precise evaluation, offering novel insights into breast cancer metastasis.

## Materials and methods

### Data sources

We obtained single-cell RNA sequencing (scRNA-seq) data from 13 breast cancer patients (16 samples), including data from primary tumors and tumors with brain, bone, and liver metastases, comprising a total of 81,834 cells. Additionally, we obtained single-cell sequencing data for lung metastatic tumors from two breast cancer patient-derived xenograft (PDX) models, which included 695 metastatic cells. All these single-cell datasets were sourced from the GEO database. Furthermore, we acquired bulk transcriptome and microarray datasets of patients with breast cancer with metastatic organ annotations from the GEO database. In addition, the data used for survival analysis were retrieved from the TCGA database (TCGA-BRCA). Table S1 provides detailed information on all the datasets.

### scRNA-seq data processing

We removed genes expressed in fewer than three cells. Cells with fewer than 200 or more than 5000 detected genes were filtered out. We applied a mitochondrial gene content threshold, removing cells with more than 20% mitochondrial gene expression.

The identification of major cell types was the initial step. Using well-established marker genes, we identified various cell types, such as T cells (e.g., CD3D, CD4, CD8A, CD3E, and CD3G), B cells (e.g., CD79A and CD19), macrophages (e.g., CD68, CD14, and CD163), epithelial cells (e.g., EPCAM and CDH1), erythrocytes (e.g., HBB, and HBA2), fibroblasts (e.g., PDGFRB, PDGFRA, COL1A1, POSTN and DCN), astrocytes (e.g., GFAP, AQP4, and S100B), and endothelial cells (e.g., CD34, VWF, and FLT1). The cellular composition within the sample was detailed through cell annotation using the Seurat R package [16]. To distinguish malignant epithelial cells from normal epithelial cells, we employed InferCNV, a method that utilizes single-cell RNA-seq data to detect large-scale chromosomal alterations typical of cancer cells. We compared the gene expression profiles of suspected malignant cells to those of a reference set of normal cells, including endothelial cells, B cells, T cells, and other non-malignant cell types. These cells were selected because they are non-cancerous and are expected to have stable chromosomal structures, making them suitable for identifying genomic abnormalities in malignant epithelial cells. Thus, we were able to infer large-scale chromosomal variations associated with tumor progression.

To explore the dynamic changes in gene expression over time and understand the trajectory of cellular differentiation, we conducted pseudotime analysis using Monocle2 (2.24.0) [17]. This tool reconstructs cell fate decisions and developmental trajectories, allowing for us to infer the temporal sequence of cellular states and identify key regulators of these processes in cancer cells.

Understanding intercellular communication is vital for deciphering the tumor microenvironment and the interactions between different cell types. We utilized CellChat (version 1.6.1) to analyze cell‒cell communication networks, aiming to unravel the differences in interactions between primary and metastatic tumors within their respective microenvironments [18]. This analysis is crucial for understanding the conditions that different organs provide for the colonization of metastatic tumor cells.

### Genomic analyses

To identify differentially expressed genes (DEGs) between primary and metastatic sites, we compared the bulk gene expression profiles of primary breast cancer tumors with those of four metastatic breast cancer tumors using the '*limma*' package [19].

Additionally, we used weighted gene co-expression network analysis (WGCNA) to identify co-expressed genes in breast cancer tumors that metastasized to the same organ [20]. We used the '*goodSamplesGenes*' function from the WGCNA package to remove abnormal genes and samples from the expression data and then cluster the samples separately. We performed hierarchical clustering of metastasis samples for each organ to detect potential outliers. Specifically, we examined the dendrogram structure and manually set the '*cutHeight*' parameter to 125, a threshold determined based on the significant distance differences between samples. Samples that formed distinct outlier branches, which were separated from the main cluster, were removed. To further refine the clustering, we used the '*cutreeStatic*' function to divide the samples into different clusters, ensuring that each cluster contained at least 10 samples so that any clearly isolated samples were classified as outliers. Following outlier removal, we used the '*pickSoftThreshold*' function to systematically compute the scale-free topology fitting index and average connectivity across different soft threshold values, allowing for us to select the optimal soft threshold power for network construction. Finally, we applied the '*blockwiseModules*' function to identify gene modules based on topological overlap, facilitating the detection of biologically relevant co-expression patterns.

GO enrichment analysis was conducted on the DEGs, identifying GO terms with p values less than 0.05. The '*enrichGO*' function from the '*clusterProfiler*' package was used, with the genome referenced via the '*org.Hs.eg.db*' package. '*clusterProfiler*' is an R package designed for functional enrichment analysis [21]. It is primarily used for functional annotation of genes and genomic data, aiding in pathway enrichment analysis to provide deeper insights into the biological functions underlying the data. Similarly, KEGG pathway enrichment analysis was performed using the '*enrichKEGG*' function, identifying pathways with p values less than 0.05.

To further investigate the impact of identified key transfer genes on patient survival, patients were divided into high-expression and low-expression groups based on the expression values of the genes in the patients. Kaplan‒Meier (K‒M) survival analysis was then performed, and survival curves for these two groups were plotted. The log-rank test was used to assess the significance of differences between the survival curves. A p value of less than 0.05 was considered indicative of a significant impact of the gene on patient survival. The survival analysis was primarily conducted using the '*survival*' and '*survminer*' packages in R.

### Lasso model for screening biomarker genes

We conducted LASSO regression by using the '*glmnet*' package and screened the genes highly correlated with tumor metastasis tendency from the identified organ-specific genes associated with breast cancer metastasis [22]. '*glmnet*' is an R package primarily used for the regularization of generalized linear models and the statistical analysis of high-dimensional data. It provides an efficient algorithm for fitting LASSO regression.

The R '*survival*' package was used to analyze the impact of a single gene on the patient survival rate, and a p value < 0.05 indicated statistical significance. The dataset from GSE9893 (n = 155) was used to train the model.

### Deep neural networks for breast cancer metastasis prediction

We integrated a set of organ-specific metastasis-associated genes from four metastatic sites and used them as characteristic markers to investigate organotropism in breast cancer. Then, we extracted the expression values of the characteristic genes from the breast cancer expression profiles. The gene expression profile data used for training and testing the DNN model were initially standardized using z scores. The data were classified into five types: primary site, liver metastasis, lung metastasis, brain metastasis, and bone metastasis.

**Fig. 1:**
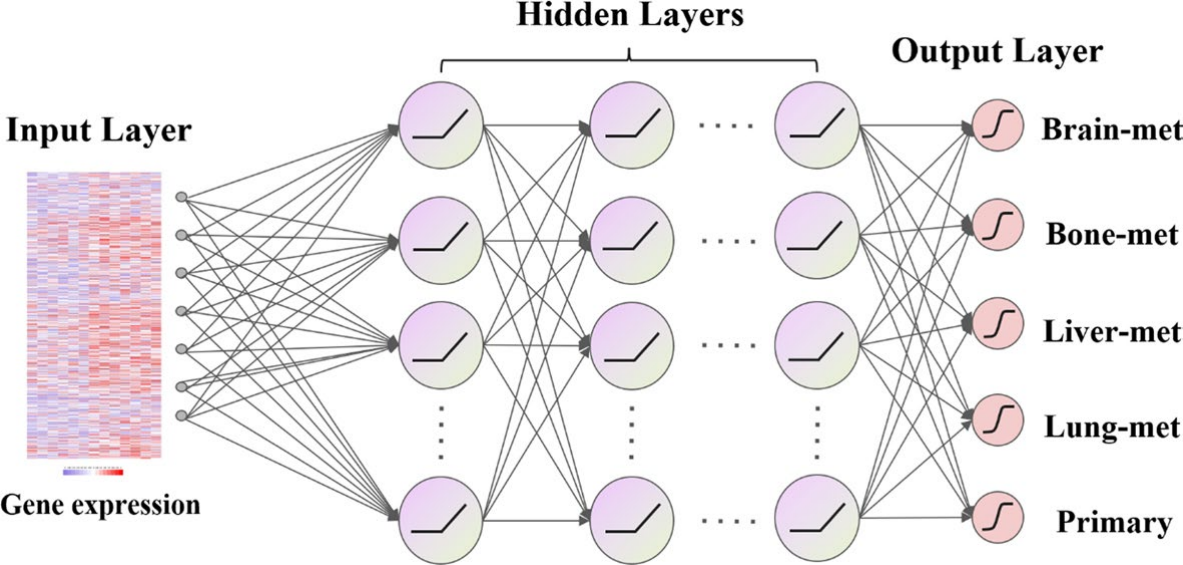
Deep neural network framework for predicting breast cancer organotropism

The DNN mainly consists of three main parts, namely, input layer, hidden layer, and output layer. The framework of the DNN is shown in Fig. 1. The number of neurons in the input layer is 1056, and the hidden layer consists of 5 fully connected layers, each with 2048 neurons. Each layer has a dropout rate of 0.5, meaning that 50% of the neurons in each layer are randomly discarded during training. This technique requires that the weights of each neuron be optimized to minimize dependency on other neurons in the same layer and to increase the robustness of the model to noise and overfitting.

In neural networks, the activation function plays a critical role in introducing nonlinearity, enabling the model to learn complex patterns. ReLU, a commonly used activation function, is favor for its computational efficiency and ability to mitigate the vanishing gradient problem. When information is output by neurons in the hidden layers, it passes through the ReLU activation function, allowing for the network to model complex nonlinear relationships. In our experiments, ReLU demonstrated the best performance, yielding the highest accuracy, precision, recall, and F1 score, making it the most suitable choice for our task. While other activation functions, such as Sigmoid, ELU, and Tanh, offer distinct properties that can influence model training and prediction outcomes, their performance was slightly inferior to that of ReLU. The number of neurons in the output layer is determined by the purpose of the experiment. We set it to 5 neurons to predict the possibility of four kinds of organ metastasis (bone, brain, liver and lung) or non-metastasis in patients with breast cancer. The output layer neurons use the sigmoid function, which scales the output values between 0 and 1, representing the high or low risk of tumor metastasis in patients (Fig. 1).

Since predicting the risk of tumor metastasis is essentially a multiclass classification problem, the loss function used for network training is categorical cross-entropy. To prevent overfitting, L2 regularization was applied to the parameters, with the following specific formula:

1 $${\text{Loss}} = - \frac{1}{N}\sum\limits_{i = 1}^{N} {\sum\limits_{j = 1}^{C} {y_{ij} } } \log (p_{ij} ) + \lambda \sum\limits_{k} {w_{k}^{2} }$$

*y*_*ij*_ is the true label of the *i*-th sample in class *j*, *p*_*ij*_ is the probability that the model predicts that the *i*-th sample belongs to class *j*, *λ* is the regularization parameter used to control the intensity of regularization, and *w*_*k*_ is the weight parameter in the model.

To comprehensively evaluate the performance of the deep learning model, we employed several standardized evaluation metrics, including accuracy, precision, recall, and the F1 score. Furthermore, both the DNN model and the SHAP package, which are used for model interpretation, were implemented in Python.

## Results

### Gene expression profiles of primary and metastatic breast cancer tumors

After the genes that were differentially expressed between metastatic and non-metastatic samples of breast cancer were identified using the limma package, we screened for genes with significant differential expression using p.adjust < 0.05 as the criterion. Furthermore, we performed differential gene expression analysis to compare the single-cell data of primary breast cancer tumors with those of other metastatic site tumor (bone, brain, liver and lung). This analysis involved identifying genes that are significantly upregulated or downregulated between the primary and metastasis groups, providing insights into the molecular differences and potential pathways driving the divergence in tumor behavior or response to treatment. The full list of differentially expressed genes is provided in Table S2. Figure S1 presents volcano plots illustrating these differentially expressed genes, with Figure S1A-D corresponding to comparisons between the primary tumor and brain, bone, liver, and lung metastases.

**Fig. 2:**
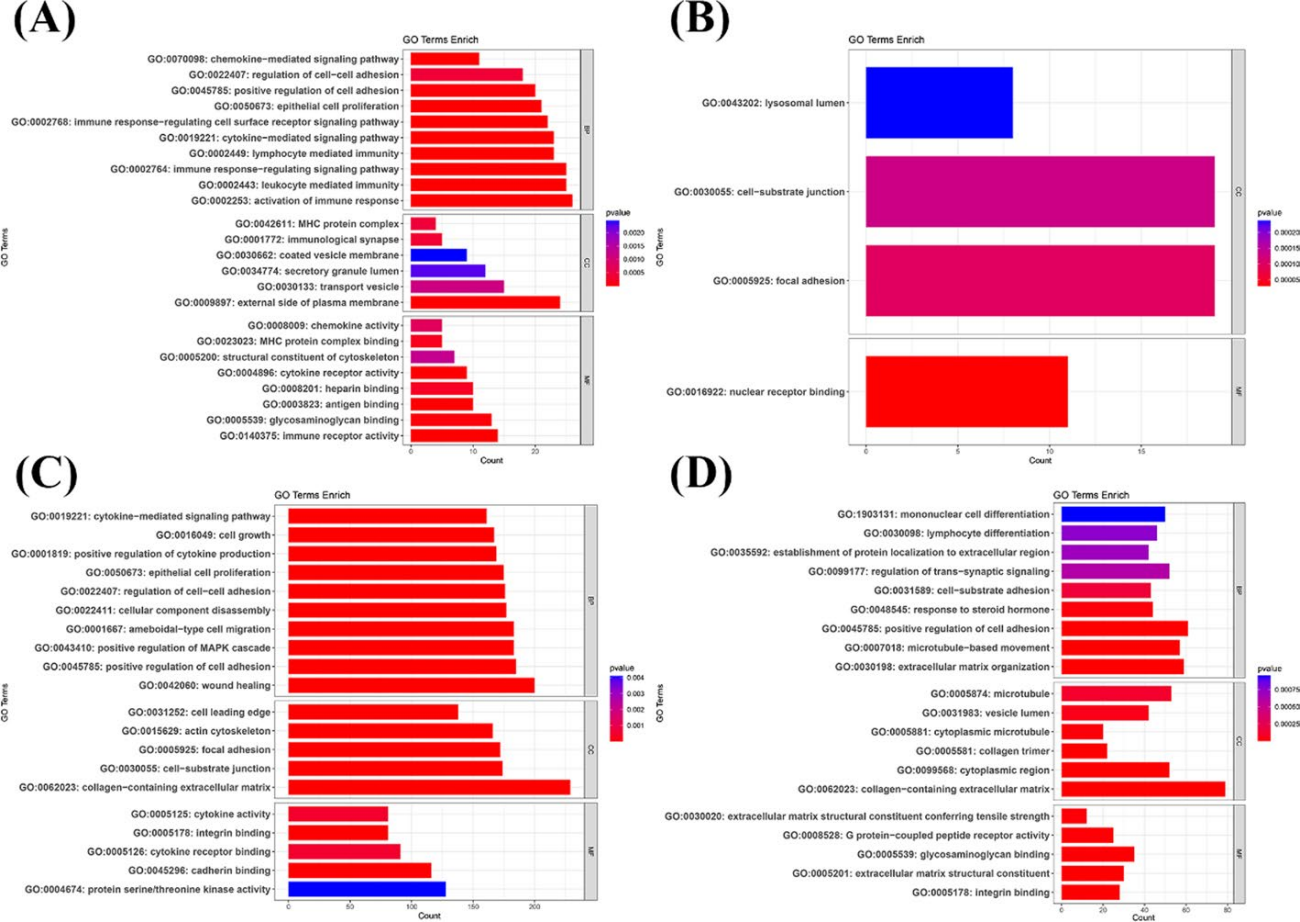
GO term enrichment analysis of four metastatic organs of breast cancer. **A** GO term enrichment analysis of bone metastasis samples. **B** GO term enrichment analysis of brain metastasis samples. **C** GO term enrichment analysis of liver metastasis samples. **D** GO term enrichment analysis of lung metastasis samples

**Fig. 3:**
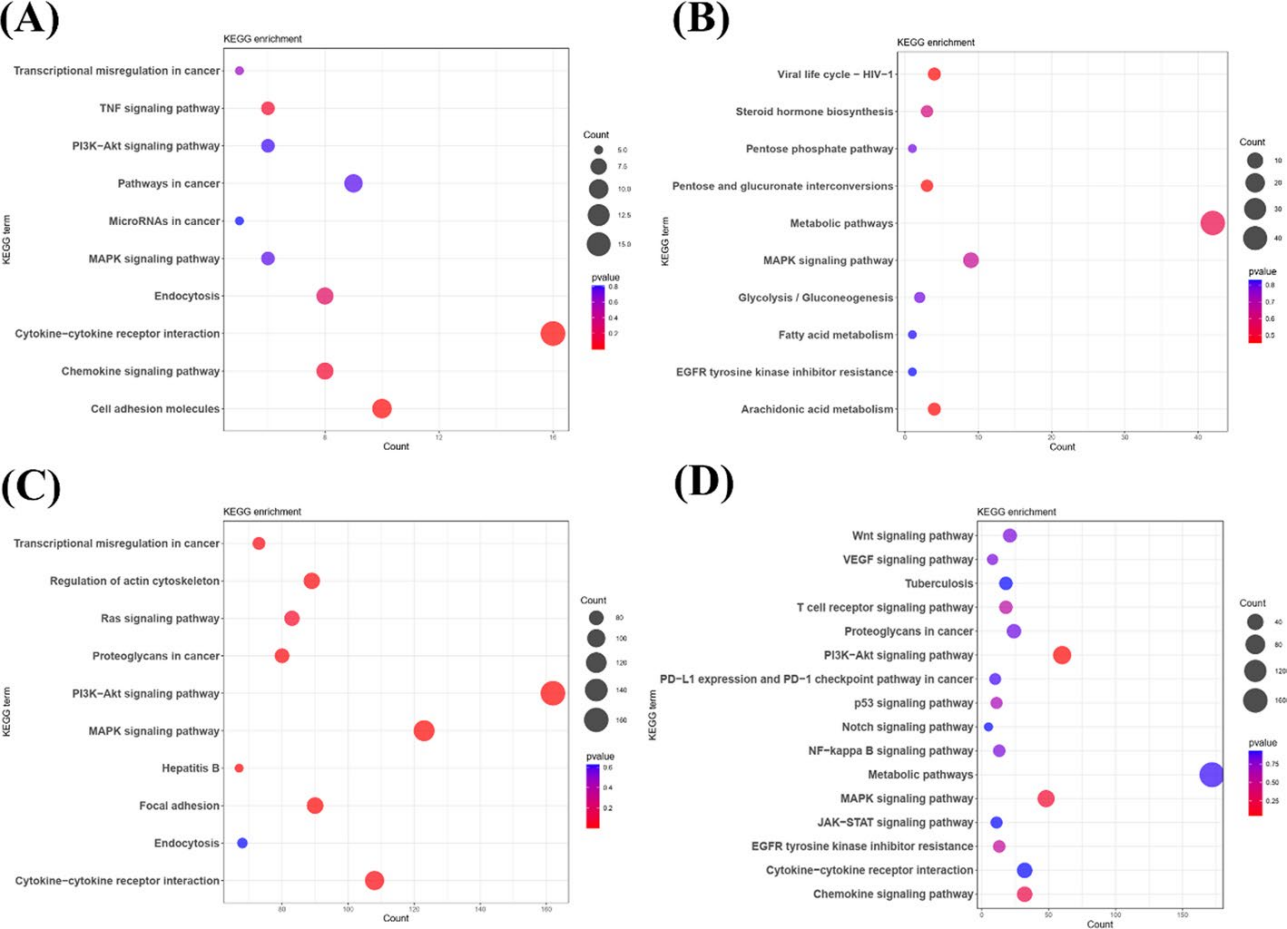
KEGG enrichment analysis of four metastatic organs of breast cancer. **A** KEGG enrichment analysis of bone metastasis samples. **B** KEGG enrichment analysis of brain metastasis samples. **C** KEGG enrichment analysis of liver metastasis samples. **D** KEGG enrichment analysis of lung metastasis samples

GO term enrichment analysis revealed that signaling pathways, such as positive regulation of cell adhesion and focal adhesion, are involved in at least two metastatic tumors. Breast cancer tumors in each metastatic organ display distinct active signaling pathways: 'activation of immune response’ in bone, ‘wound healing’ in the liver, and 'cell–substrate adhesion’ in the lung. KEGG enrichment analysis revealed that the MAPK signaling pathway, metabolic pathways, and PI3K-Akt signaling pathway are commonly activated across all metastatic sites. Additionally, specific signaling pathways that are active in each metastatic organ include the chemokine signaling pathway in bone, the Ras signaling and hepatitis B pathways in the liver, the chemokine signaling pathway in the lung, and arachidonic acid metabolism and steroid hormone biosynthesis in the brain. Figure 2 and Fig. 3 illustrate the results of the enrichment analysis.

**Fig. 4:**
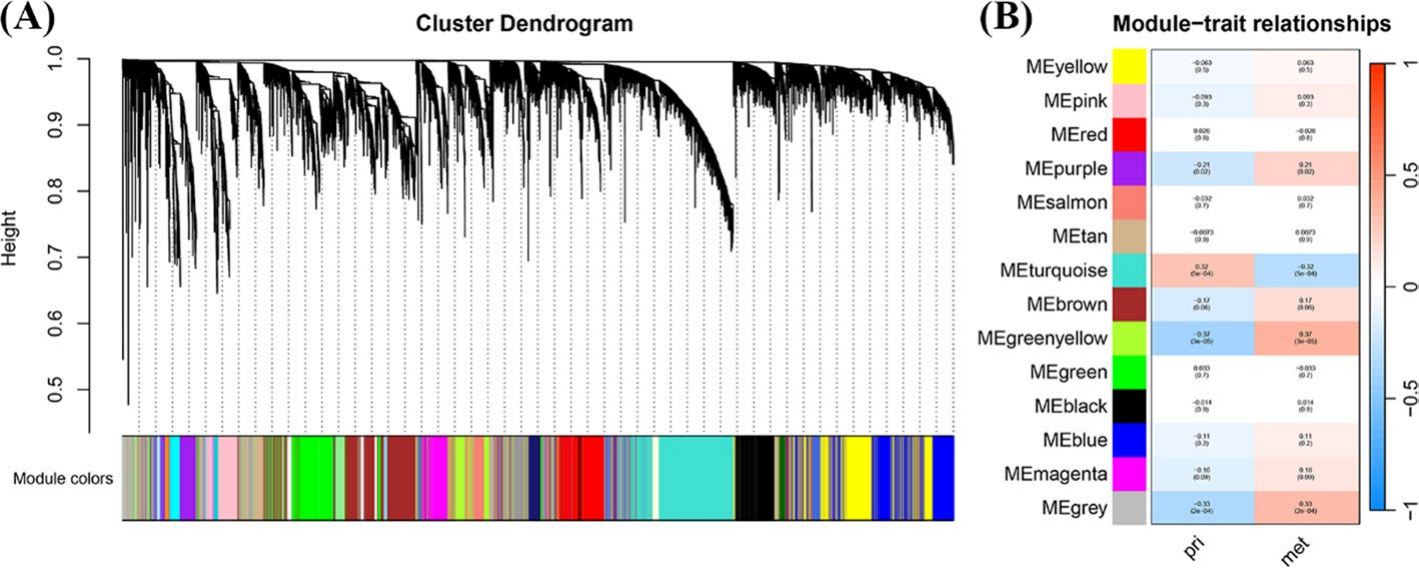
Recognition of WGCNA modules in primary and metastatic breast cancer tumors. **A**, **B** The heatmap shows the correlation between gene modules and traits

To further identify the differences between primary and metastatic breast cancer tumors (metastatic sites were not available), we conducted differential gene analysis based on the GSE9893 dataset and performed WGCNA on metastatic breast cancer samples. After the expression profiles of the primary breast cancer tumor samples were analyzed by WGCNA, a total of 14 modules were obtained. The WGCNA results for the metastatic tumor are shown in Fig. 4. Figure S2 shows the sample clustering dendrogram for outlier detection in the WGCNA.

We compared the co-expressed gene sets identified by WGCNA with the differentially expressed gene sets of primary tumors, metastatic tumors (site unknown), and tumors from four metastatic organs. These gene sets share partially intersecting genes, which are shown in Figure S3. We used all the intersecting genes (GeneSet1, Table S3), for a total of 87 genes, as the base gene set for the subsequent LASSO screening.

### Lasso screening

**Fig. 5:**
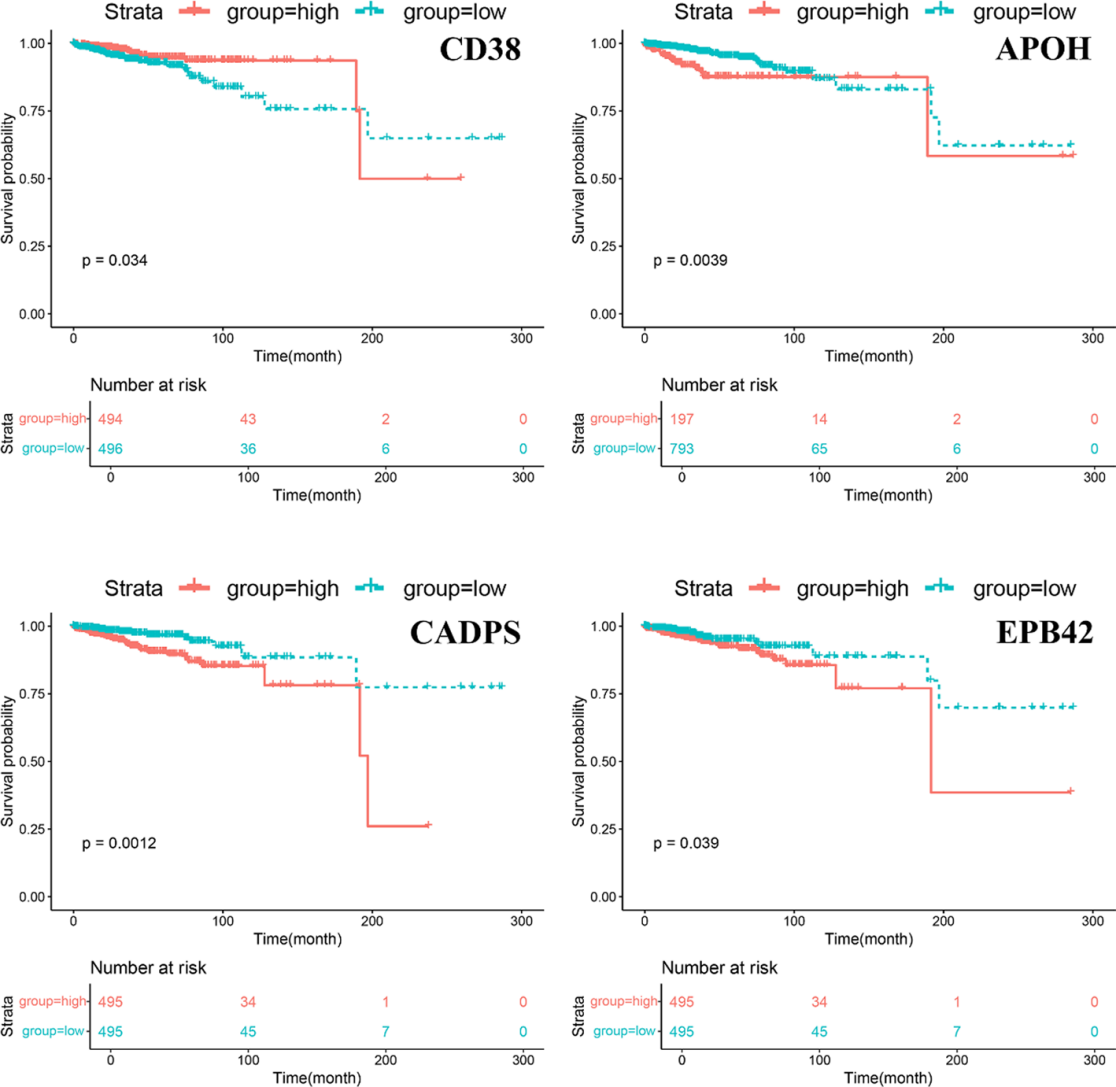
Kaplan–Meier survival curves of four key genes screened by Lasso for breast cancer metastasis

To explore the relationship between metastasis-related genes and breast cancer prognosis, we used GeneSet1 and a Lasso model to screen for specific metastasis genes that impact the survival outcomes of breast cancer patients. We performed overall survival (OS) analysis, and the results of survival analysis revealed that CD38, APOH, EPB42, and CADPS had significant impacts on breast cancer patients; the survival curves are shown in Fig. 5, with p values from the log-rank test of 0.034, 0.0039, 0.039, and 0.0012, respectively. The hazard ratios (HRs) for each gene were as follows: CD38: HR = 0.973, APOH: HR = 1.167, CADPS: HR = 1.001, and EPB42: HR = 2.266. The results of the LASSO regression analysis are listed in Table S3.

LASSO regression analysis effectively reduces the number of genes under consideration by selecting the most relevant features. This approach enabled us to refine the high-dimensional gene expression dataset into a smaller subset of genes that are strongly associated with metastasis, thereby enhancing the interpretability of our findings. Furthermore, the LASSO regression results provide insights into the relationship between gene expression and metastasis. Genes with positive coefficients may be directly associated with metastatic potential, whereas genes with negative coefficients could indicate a compensatory mechanism in which the body attempts to suppress metastasis.

### Characteristics of the tumor microenvironment at breast cancer metastasis sites

**Fig. 6:**
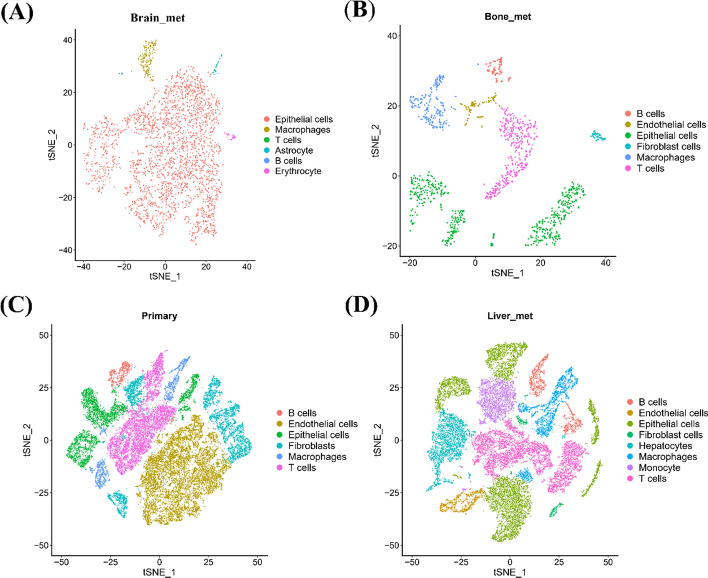
Identification of cell subsets in primary and metastatic sites of breast cancer. **A** tSNE plot of single cells from brain metastasis samples, colored by major cell types. **B** tSNE plot of single cells from bone metastasis samples, colored by major cell types. **C** tSNE plot of single cells from primary tumor samples, colored by major cell types. **D** tSNE plot of single cells from liver metastasis samples, colored by major cell types

Through single-cell RNA sequencing analysis of metastatic breast cancer cells in the brain, we identified six distinct cell types: astrocytes, B cells, epithelial cells, erythrocytes, macrophages, and T cells (Fig. 6A). Similarly, at both the primary and bone metastasis sites, we observed six cell types, including macrophages, epithelial cells, endothelial cells, B cells, fibroblasts, and T cells (Fig. 6B and C). Figure S4 presents a tSNE plot showing that only epithelial cells were annotated in the single-cell data of lung metastases.

The observation of a large population of epithelial cells in the single-cell annotation results for brain metastasis is indeed an interesting finding. This may be significantly influenced by the specific tumor types and the nature of the samples selected. The relative scarcity of other cell types in the annotation could suggest that the brain metastases in our samples were primarily composed of tumor cells, with limited infiltration by immune cells, stromal cells, or other supportive cell types. This may reflect the early stages of metastatic colonization, where epithelial cells represent the predominant population.

**Fig. 7:**
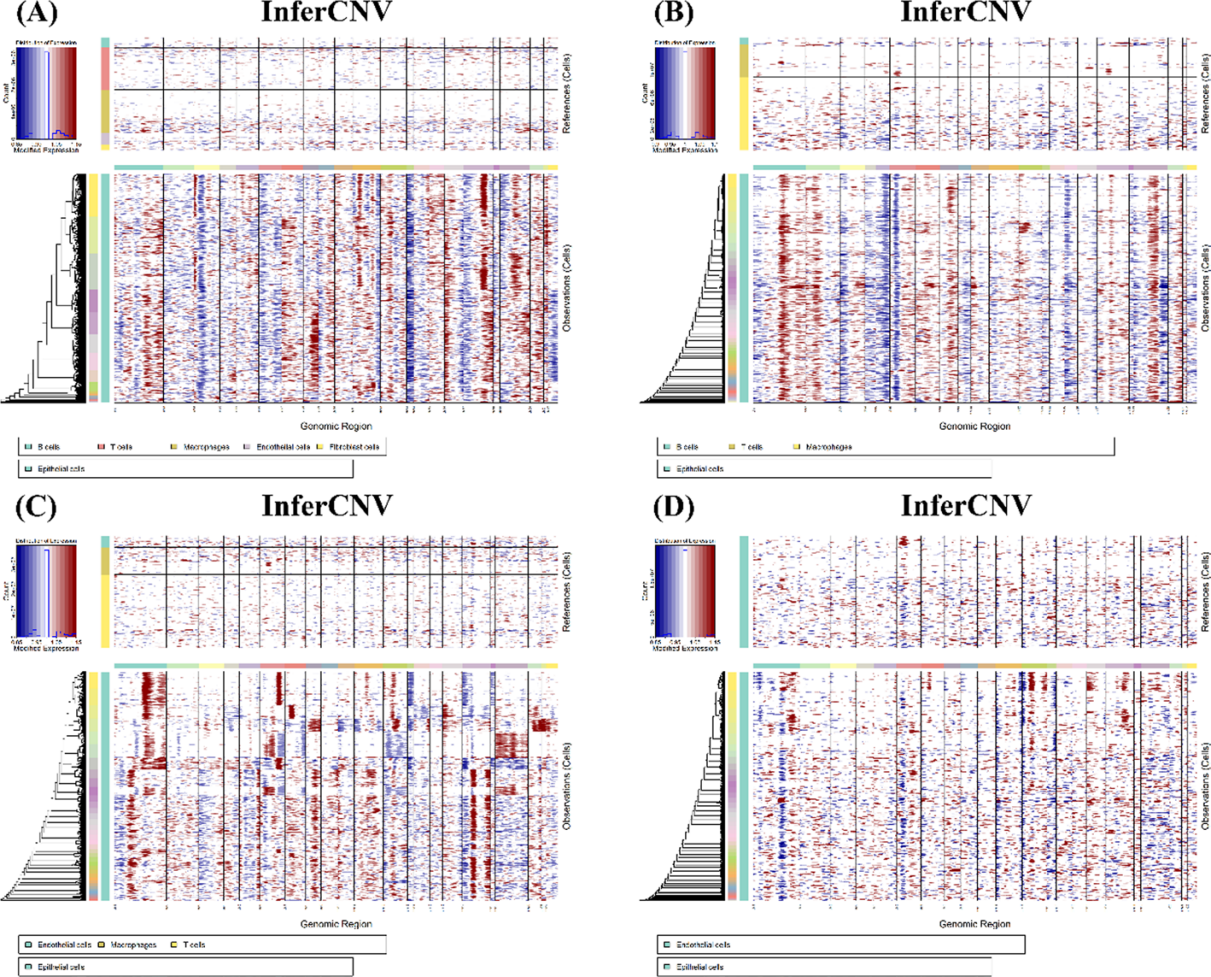
CNV maps of primary and metastatic breast cancer locations, highlighting chromosomal amplifications (red) and deletions (blue). **A** InferCNV of bone metastasis in breast cancer. **B** InferCNV of brain metastasis in breast cancer. **C** InferCNV of liver metastasis in breast cancer. **D** InferCNV of primary tumor in breast cancer

Then, utilizing inferCNV, we successfully identified malignant tumor cells originating from epithelial cells. Using annotated normal cells (such as endothelial and immune cells) as a reference, we identified tumor cells based on their CNV profiles. As shown in Fig. 7, normal reference cells presented relatively stable CNVs, with minimal changes in gene copy numbers. In contrast, malignant epithelial cells show substantial alterations in CNVs, including gene amplifications and deletions, which are visually represented in red and blue, respectively. These CNV changes are evident across different chromosomes, underscoring the genomic instability of tumor cells.

We subsequently compared the differential gene expression profiles of these malignant tumor cells across different organs (Fig. 7). We clearly observed that, compared with malignant epithelial cells at the primary site, chromosomal loss and amplification are more pronounced in malignant epithelial cells from metastatic tumor sites.

**Fig. 8:**
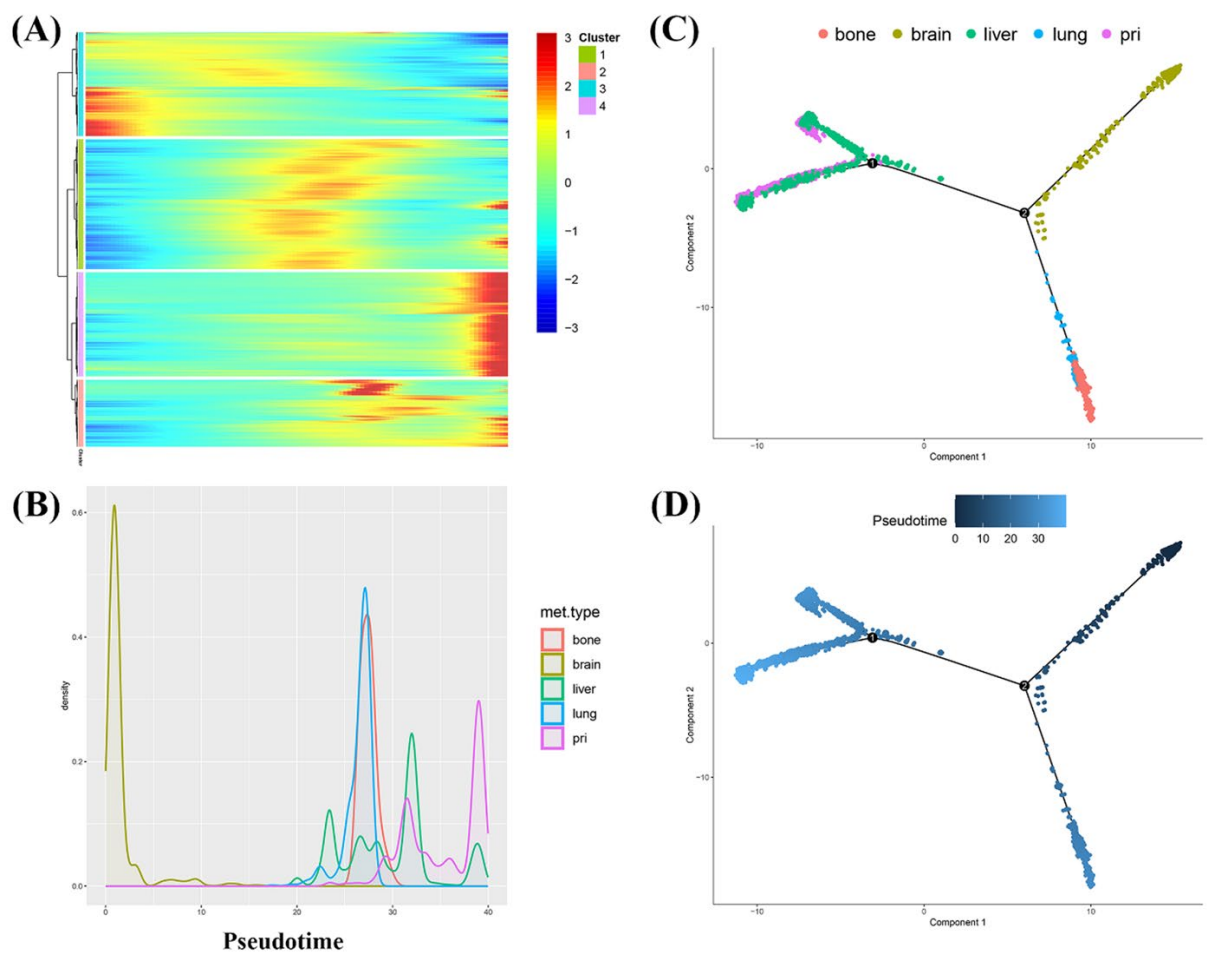
The trajectory illustrates the dynamic progression of malignant tumor cells. **A** Heatmap showing the expression of representative genes across single cells along the pseudotime trajectory, grouped by gene clusters. **B** Pseudotime density plot depicting the distribution of malignant tumor cells from different organs (bone, brain, liver, lung, and primary) along the pseudotime axis. **C** Simulation of the development trajectory of malignant cells, colored by metastasis sites. **D** Simulation of the development trajectory of malignant cells, colored by development stage

We employed Monocle2 to perform a pseudotime analysis of malignant epithelial cells from primary and metastatic breast cancer, aiming to explore the dynamic cellular changes during the progression from the primary tumor to metastatic lesions. A total of 9059 genes associated with dynamic changes in cellular behavior were identified and can be classified into four categories (Fig. 8A). Some genes exhibited significant fluctuations in expression levels over time, as observed in Cluster 1 and Cluster 2 in Fig. 8A. The expression levels of other genes gradually increased over time (Cluster 3), whereas those of some genes gradually decreased (Cluster 4). In summary, pseudotime analysis revealed distinct gene expression patterns with temporal regularity.

The density map shown in Fig. 8B corresponds to the temporal sequence presented in Fig. 8A. We observed that primary malignant epithelial cells appeared at the early stage of the pseudotime trajectory, whereas malignant epithelial cells from brain metastases emerged at the late stage. These findings suggest that malignant epithelial cells from brain metastases exhibit notable heterogeneity compared with those from the primary breast cancer site. This pattern is more pronounced in Fig. 8C. Figure 8C, which illustrates the specific positioning of malignant epithelial cells from breast cancer within the pseudotime trajectory, highlighting their distribution across both primary and metastatic organs. We can clearly observe that the temporal heterogeneity between malignant epithelial cells in liver metastases and those at the primary site is relatively small. Malignant epithelial cells in lung and bone metastases exhibited temporal continuity, whereas those in brain metastases showed a distinct divergence from other metastatic sites. Figure 8D illustrates the pseudotime progression of malignant epithelial cells across different metastatic sites, represented by varying shades of color. These results suggest that liver metastases may arise at an earlier stage of disease progression, whereas brain metastases are more likely to occur at a later stage.

### Differences in the tumor microenvironment between primary and metastatic sites of breast cancer

The tumor microenvironment is a complex ecosystem that includes various cell types that interact with each other, collectively influencing tumor growth, invasion, and metastasis. In breast cancer, the tumor microenvironment plays a crucial role in tumor initiation, progression, and metastasis. We found that the microenvironment at the primary tumor site primarily comprises tumor cells, fibroblasts, and immune cells. In contrast, the microenvironment at metastatic sites not only includes immune cells and tumor cells but also features stromal and parenchymal cells unique to each organ. For example, the brain contains astrocytes, whereas the liver contains hepatocytes.

**Fig. 9:**
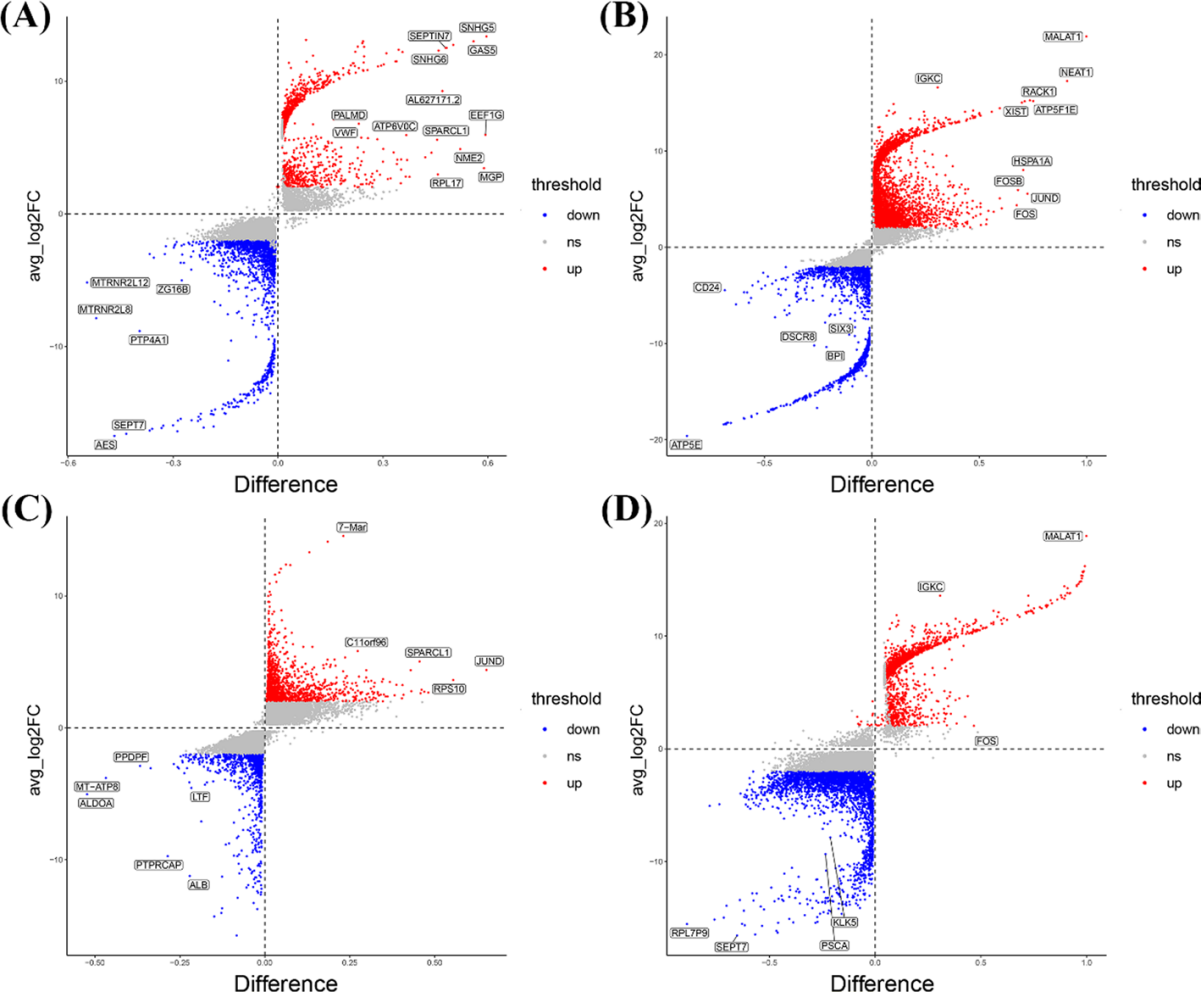
Differential gene expression analysis of malignant cells between primary and metastatic sites based on single-cell RNA sequencing. **A** Comparison of single-cell differentially expressed genes between primary breast cancer and bone metastasis. **B** Comparison of single-cell differentially expressed genes between primary breast cancer and brain metastasis. **C** Comparison of single-cell differentially expressed genes between primary breast cancer and liver metastasis. **D** Comparison of single-cell differentially expressed genes between primary breast cancer and lung metastasis

We compared the differentially expressed genes between primary breast cancer and four metastatic sites (bone, brain, liver, and lung) using single-cell RNA-seq data. By analyzing the differentially expressed genes, we can gain deeper insights into the molecular mechanisms underlying breast cancer progression from the primary site to various metastatic locations. The genes differentially expressed between tumor cells in the four metastatic organs and those in the primary site are listed in Table S4. Figure 9 presents the results of the single-cell RNA sequencing analysis, which identified the genes that were differentially expressed between primary and metastatic breast cancer cells. The figure highlights distinct gene expression profiles across the four metastatic sites (bone, brain, liver, and lung), revealing site-specific molecular signatures.

**Fig. 10:**
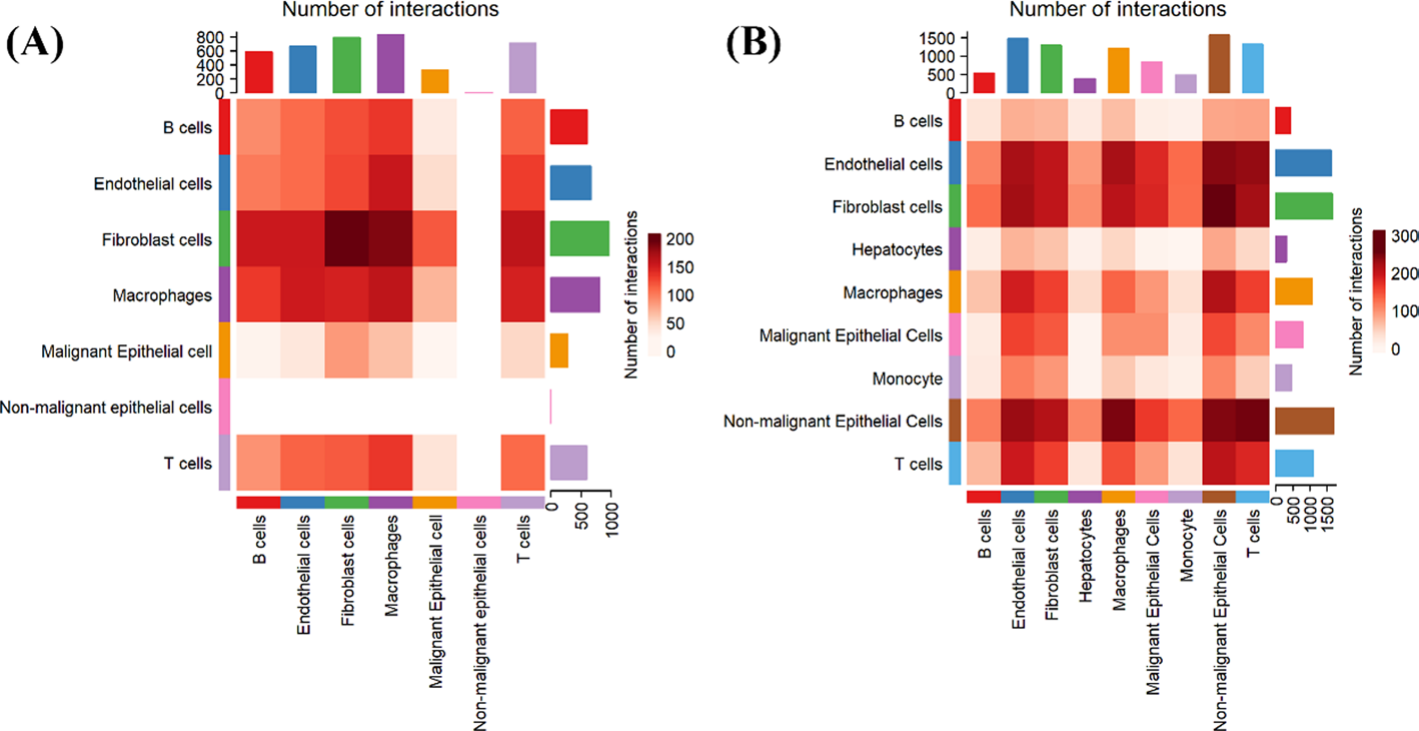
Cell type communication intensity in the microenvironment of breast cancer metastases. **A** Heatmap of communication intensity between cell types in the bone metastasis tumor microenvironment of breast cancer. **B** Heatmap of communication intensity between cell types in the liver metastasis tumor microenvironment of breast cancer

We utilized CellChat to analyze cell communication within the tumor microenvironments of both primary and metastatic breast cancer sites. In primary tumors, communication between malignant epithelial cells and T cells was relatively weak. However, at metastatic sites, this interaction was significantly enhanced. Additionally, there was a strong interaction between malignant tumor cells and stromal cells in metastatic organs (fibroblasts in the bone and liver), as shown in Fig. 10.

Since only one type of epithelial cell was annotated in breast cancer with lung metastasis, cell communication analysis could not be performed. This is because the single-cell RNA data for lung metastasis were derived from PDX models, where tumor-associated stromal and immune cells are largely replaced by mouse-origin cells. Consequently, our dataset contains primarily human tumor cells and lacks human-derived stromal or immune components. Therefore, CellChat, which analyzes cell communication through ligand‒receptor interactions, cannot be applied to analyze the lung metastatic tumor microenvironment.

**Fig. 11:**
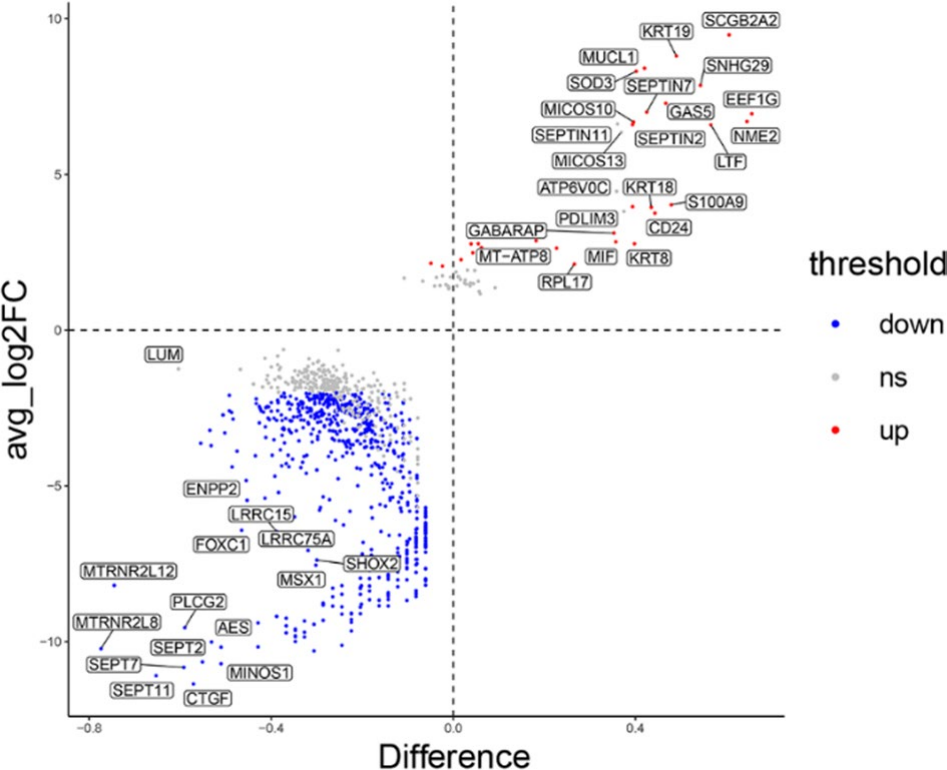
Comparison of differentially expressed genes in fibroblasts between bone and liver metastases of breast cancer

On the basis of the results of the pseudotime analysis, we speculated that fibroblasts play a key role in the organ-specific metastasis of breast cancer. To further explore this, we compared the differentially expressed genes in fibroblasts between bone and liver, as shown in Fig. 11.

### Deep learning model for predicting breast cancer metastatic patterns

**Fig. 12:**
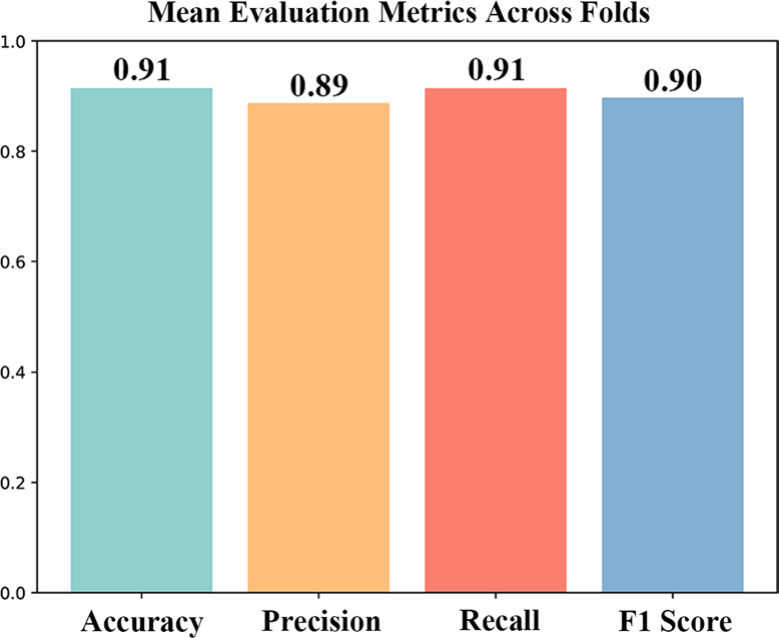
Evaluation of the accuracy of the DNN prediction model for organotropism in breast cancer metastasis

Our deep learning model achieved an accuracy of 0.91, indicating its strong overall performance in correctly classifying metastasis risk. With an F1 score of 0.9, our model balances precision and recall effectively, highlighting its robustness in handling both false positives and false negatives. The precision of 0.89 and recall of 0.91 further demonstrate the ability of this DNN model to accurately identify true positive cases and capture most actual metastatic instances. The results are shown in Fig. 12. The evaluation results of the other activation functions are shown in Figure S5.

**Fig. 13:**
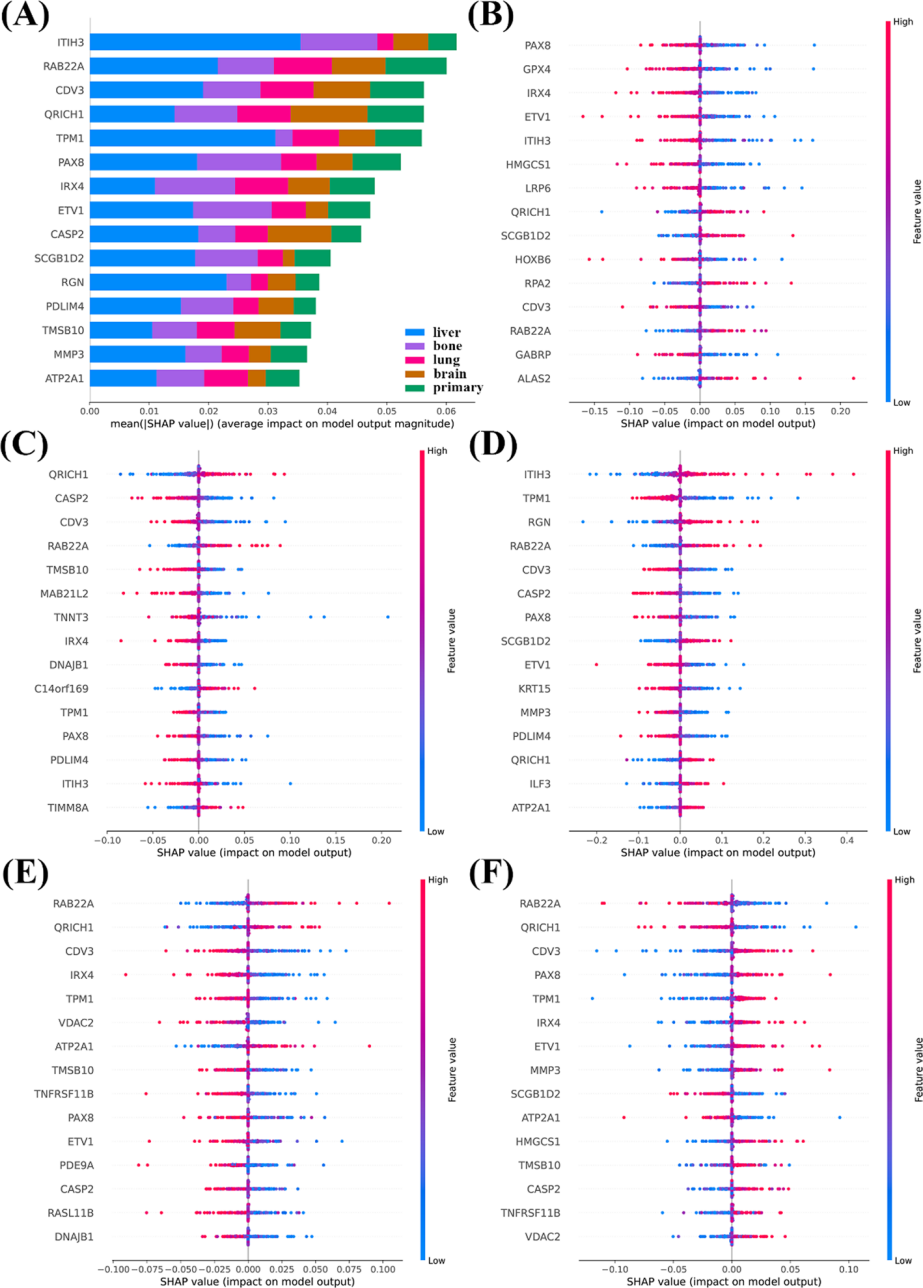
SHAP analysis of DNN model. **A** SHAP value ranking of gene features across five classification groups. Bar plot showing the total SHAP values for the top-ranked gene features across five categories: liver metastasis, bone metastasis, lung metastasis, brain metastasis, and primary breast cancer (non-metastasis). SHAP values indicate the contribution of each gene to the prediction model for organotropism. **B** SHAP value ranking of bone metastasis gene features. **C** SHAP value ranking of brain metastasis gene features. **D** SHAP value ranking of liver metastasis gene features. **E** SHAP value ranking of lung metastasis gene features. **F** SHAP value ranking of primary site gene features

To ensure the transparency and interpretability of this model, we utilized SHAP values. SHAP analysis provided insights into the contribution of each feature to the predictions, allowing for us to understand the biological significance behind the metastatic behavior of breast cancer. The top 15 ranked feature genes, based on their total SHAP value scores, are presented in Fig. 13. The interplay between these biomarkers likely drives the complex process of breast cancer metastasis, emphasizing the importance of a multifaceted approach in predicting and managing metastatic risk.

## Discussion

Our study of breast cancer metastasis revealed distinct organ-specific patterns. By incorporating both bulk and single-cell RNA sequencing data, we identified a set of genes significantly associated with metastasis to various organs. We compared the differentially expressed genes between the four types of organ metastases and primary breast tumors and identified genes such as COL11A1, MMP11, and THBS2 as more frequently differentially expressed in metastatic sites. This observation suggests that these genes may play a universal role in the metastatic process. However, it is also possible that their differential expression is influenced by the immune microenvironment of the metastatic organs, which could contribute to suppressing or promoting tumor growth. Importantly, we focused more on genes that exhibit organotropism in breast cancer metastasis than on those that generally facilitate metastasis.

Our bulk RNA-sequencing analysis revealed several genes that may be associated with organ-specific metastasis in breast cancer. For example, CD44 and ABCC5 are highly expressed in breast cancer with bone metastasis and can serve as potential markers for this metastatic site. Previous studies have demonstrated a significant association between CD44 and ABCC5 and bone metastasis, further supporting their role in the metastatic process [23–25]. POSTN and APOH are highly expressed in tumors with liver metastasis. Previous studies have also reported a significant correlation between these two biomarkers and liver metastasis in patients with cancer [26, 27]. Unfortunately, there is currently no biological experimental research supporting the other discovered genes. Considering the biological function of the gene itself, we believe that CTBP1 can promote the brain metastasis of breast cancer, but there is limited research on this topic. Compared with tumors at the primary site, breast cancer tumors that metastasize to the lung show greater expression of genes that are more closely related to lung-specific functions, such as SFTPB and SFTA2 [28, 29]. We speculate that when tumor cells metastasize to the lungs, they must adapt to the new microenvironment to survive and grow. Tumor cells may do this by upregulating genes related to lung function, which helps them better adapt to their new environment.

These results suggest that the organ-specific metastasis of tumors is not driven by a single gene but rather by a combination of genes with diverse functions. Gene enrichment analysis also confirmed this finding. GO enrichment analysis revealed that positive regulation of cell adhesion and focal adhesion is active in the tumor cells of multiple metastatic organs. This highlights cell adhesion as a crucial factor in tumor colonization of distant organs, which is consistent with previous research on metastasis [30, 31]. Compared with metastasis to other organs, bone metastasis in breast cancer specifically triggers the activation of the immune response. In cases of breast cancer with liver metastasis, the wound healing pathway represents a relatively unique signaling mechanism. We hypothesize that this may be related to inflammatory genes involved in the wound healing pathway. Persistent inflammation can lead to changes in the organ microenvironment, potentially promoting tumor cell colonization. This hypothesis is consistent with emerging evidence that chronic inflammation can create a protumorigenic niche [32, 33]. Further studies are needed to delineate the exact molecular mechanisms by which the wound healing pathway contributes to liver metastasis.

The enrichment of the MAPK signaling pathway, metabolic pathways, and PI3K-Akt signaling pathway identified by KEGG analysis as common features in breast cancer metastasis to various organs suggests that these pathways play fundamental roles in the metastatic process. MAPK signaling is crucial for cell proliferation, differentiation, and survival, enabling cancer cells to adapt to different microenvironments during metastasis [34, 35]. Metabolic pathways are often reprogrammed in cancer cells to meet the increased energy demands and biosynthetic needs required for sustained growth and colonization in new tissues [36]. The PI3K-Akt signaling pathway is essential for promoting cell survival, growth, and migration, thereby facilitating the invasive and metastatic capabilities of breast cancer cells [37].

KEGG enrichment analysis revealed that breast cancer metastasis to specific organs is associated with distinct signaling pathways. In liver metastasis, the enrichment of the Ras signaling pathway and the hepatitis B pathway suggests that Ras-mediated cell proliferation and chronic liver inflammation increase the susceptibility of the liver to metastatic colonization. For brain metastasis, the enrichment of arachidonic acid metabolism and steroid hormone biosynthesis pathways suggests that these metabolic processes may modulate the inflammatory and hormonal environments in the brain, thereby promoting the survival and proliferation of metastatic breast cancer cells. Arachidonic acid and steroid hormones have been shown to modulate the permeability of the blood‒brain barrier, which may facilitate the infiltration of metastatic tumor cells into the brain [38, 39].

Through pseudotime analysis of single-cell sequencing data, we identified a high degree of similarity between malignant liver tumor cells and primary tumor cells, whereas malignant tumor cells in bone presented more advanced phenotypic characteristics. Moreover, the significant interactions between fibroblasts and tumor cells (including malignant and nonmalignant cells) suggest that fibroblasts play a crucial role in tumor metastasis, particularly in the regulation of tumor cell colonization by the tumor microenvironment. To further elucidate the functional differences of fibroblasts in distinct metastatic organs, we compared the differential gene expression profiles of fibroblasts in the bone and liver. Compared with those in fibroblasts in bone, genes such as KRT19, S100A9, and SEPTIN were highly expressed in fibroblasts from the liver, whereas genes such as CTGF and FOXC1 were expressed at lower levels. Previous studies have demonstrated that S100A9, FOXC1 and CTGF are involved in the molecular mechanisms underlying organ-specific metastasis in breast cancer [40–44].

Single-cell RNA sequencing analysis revealed significant heterogeneity in the tumor microenvironment of metastatic breast cancer across distinct organ sites. The patterns of cell‒cell interactions varied considerably among metastatic organs. For example, malignant epithelial cells in the brain have strong interactions with astrocytes, whereas those in the bone have more pronounced interactions with fibroblasts. This difference may be attributed to the inherent characteristics of each organ. Astrocytes are unique to the brain, highlighting organ-specific variations in cell‒cell interactions between malignant epithelial cells and resident stromal cells, such as astrocytes in the brain and fibroblasts in the bone. These interactions underscore the critical role of the local microenvironment in shaping metastatic behavior. In the brain, the strong interaction between malignant epithelial cells and astrocytes may be driven by the unique supportive role that astrocytes play in maintaining the blood‒brain barrier and neural homeostasis. Tumor cells may exploit these interactions to adapt to the microenvironment of the brain, facilitating their survival and proliferation through astrocyte-mediated signaling pathways that protect against oxidative stress or promote neural tissue remodeling. In bone, the strong interactions between malignant epithelial cells and fibroblasts could be explained by the role of fibroblasts in remodeling the extracellular matrix (ECM) and contributing to the formation of the premetastatic niche. The differential interactions observed may also reflect the varying degrees of stromal cell plasticity in response to metastatic cancer cells, with the unique cellular composition of each organ providing distinct selective pressures. Understanding these specific interactions could reveal novel therapeutic targets, as disrupting the communication between malignant epithelial cells and key stromal cells such as astrocytes or fibroblasts might inhibit the establishment and growth of metastases in these organs.

Building upon the biological insights gained from bulk and scRNA-seq analyses of breast cancer metastasis mechanisms, the integration of these data into DNN models offers a promising approach for classifying metastatic patterns and enhancing personalized treatment strategies. Our deep learning model for predicting breast cancer metastatic patterns demonstrated promising accuracy and reliability, as evidenced by its high accuracy (0.91), F1 score (0.9), precision (0.89), and recall (0.91). However, several aspects merit further discussion and investigation. First, the high performance of our model suggests that it could be a valuable tool for clinical decision-making, assisting in early intervention. Accurate predictions of metastasis risk can significantly impact patient outcomes by allowing for tailored treatment strategies. Despite the strong performance metrics, it is crucial to validate the model across diverse patient populations and clinical settings to ensure its robustness and generalizability. Second, the use of SHAP values to explain the predictions of the model enhances its transparency, providing clinicians with insights into the factors influencing metastasis risk. This interpretability is crucial for gaining clinical trust and facilitating the integration of the model into routine practice. Future work should continue to focus on improving interpretability and exploring other explainability techniques to ensure that the decisions of the DNN model are comprehensible and actionable. ITIH3, RAB22A, and CDV3 were the top three genes identified in our SHAP analysis. While RAB22A has been confirmed to be associated with breast cancer metastasis, few studies have investigated ITIH3 and CDV3 [45, 46]. However, our results indicate that these genes, with high SHAP values, are significantly associated with organ-specific breast cancer metastasis, warranting further research and exploration.

Despite the strengths of the model, several limitations must be acknowledged. The current approach may be constrained by the size and diversity of the training dataset. Additionally, while the model incorporates key genetic markers, it does not account for other important factors, such as environmental influences and lifestyle factors. A major limitation is that the model relies on genomic data from metastatic sites without incorporating paired genomic data from both primary tumors and metastatic sites, restricting its ability to predict metastasis based on primary cancer characteristics. Future studies should explore this approach to improve the clinical relevance of deep learning models for metastasis prediction.

## Conclusions

In conclusion, our findings provide a detailed genetic landscape of breast cancer metastasis, highlighting specific genes associated with organotropism. The intersection of these genes within shared GO and KEGG pathways underscores the complexity of metastatic progression and suggests potential therapeutic targets. By understanding the genetic underpinnings of organ-specific metastasis, we can develop more effective strategies for preventing and treating metastatic breast cancer.

The influence of stromal cells (fibroblasts) in target organs on tumor colonization is crucial and serves as a key determinant of tumor organotropism. Moreover, the adhesion of cells is a necessary condition for tumor metastasis and colonization. The inherent function and characteristics of the target organs themselves also significantly impact the organotropism of breast cancer tumors.

Furthermore, we developed a deep learning model based on DNN to identify key gene features associated with organ-specific metastasis in breast cancer. While the model provides valuable insights into the molecular landscape of metastasis, its primary function is to highlight genes with potential roles in metastatic progression rather than to predict organ-specific metastasis. These top gene features may provide new insights into the underlying mechanisms of organotropism and contribute to the development of targeted therapies.

## Supplementary Information

Supplementary material 1.

Supplementary material 2.

Supplementary material 3.

Supplementary material 4.

Supplementary material 5.

Supplementary material 6.

Supplementary material 7.

Supplementary material 8.

Supplementary material 9.

## Data Availability

The datasets analyzed in the current study are accessible in the TCGA and GEO databases.
