# Supplementary figures and images for: Mechanisms of organotropism in breast cancer and predicting metastasis to distant organs using deep learning
Source: Discov Oncol. 2025 Jun 11;16:1056. doi: 10.1007/s12672-025-02905-5 (PMC12158884; doi:10.1007/s12672-025-02905-5)

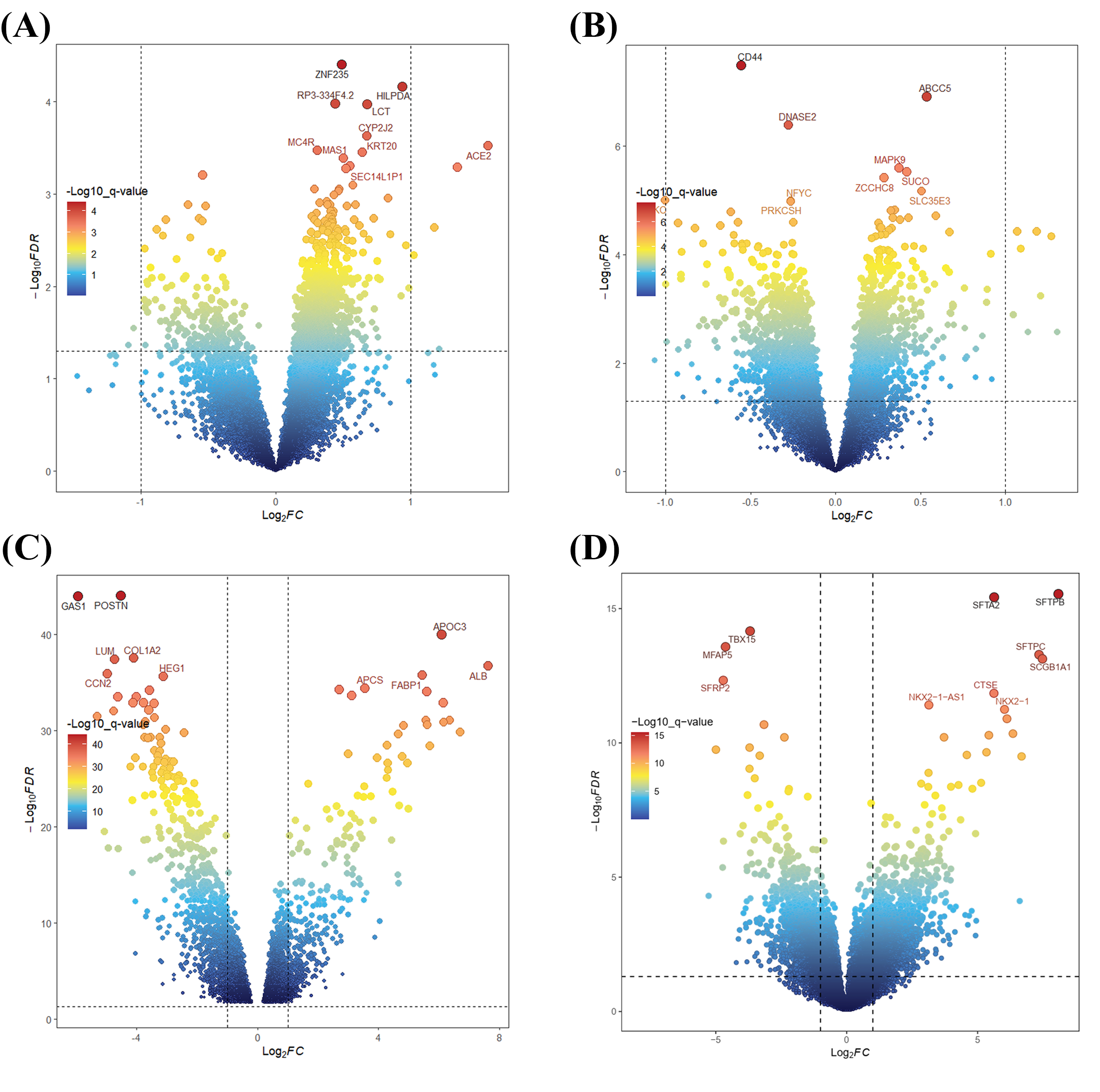

Supplement: Supplementary file 1 — Supplementary material 1. [file 12672_2025_2905_MOESM1_ESM.png]

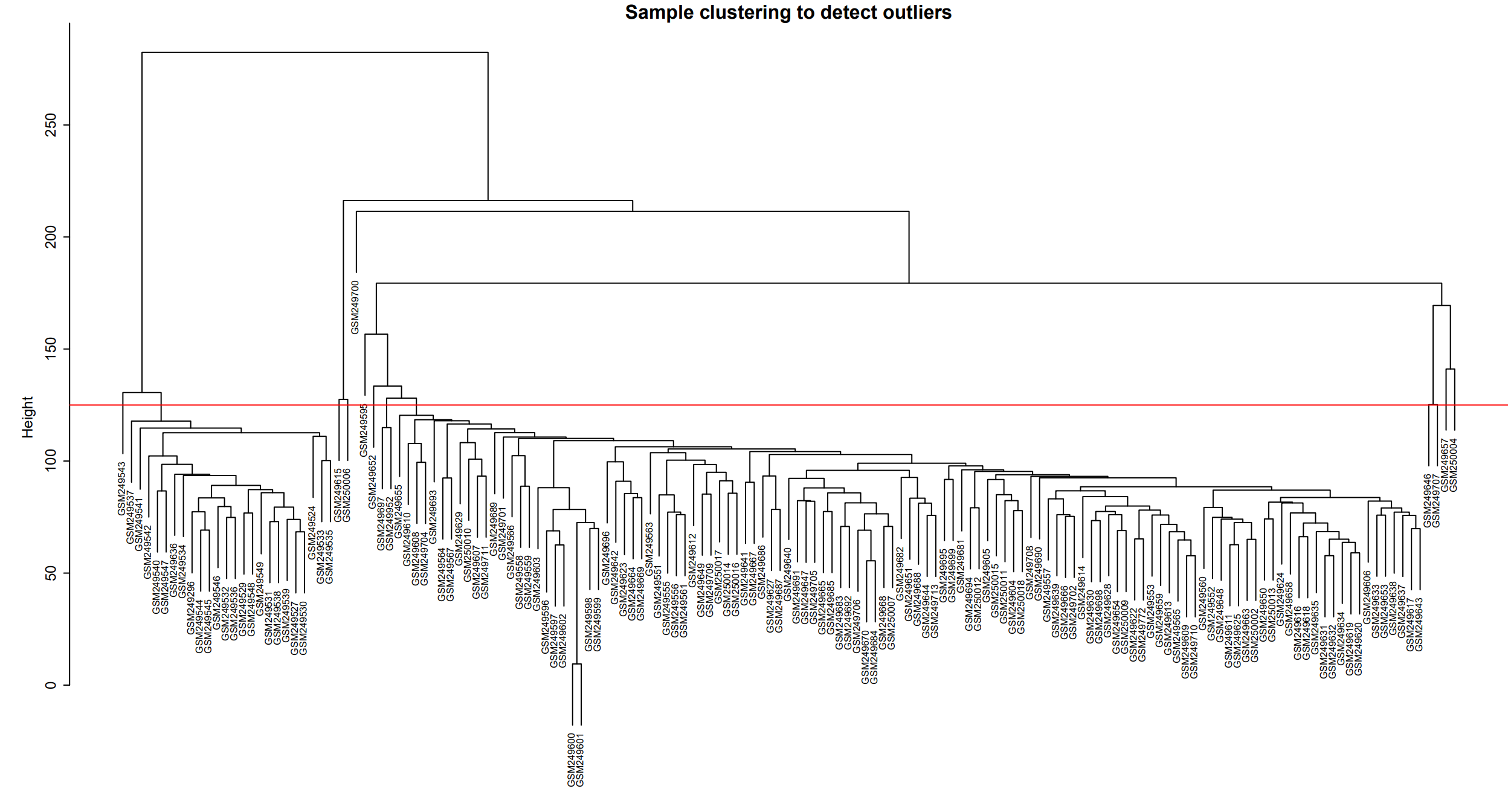

Supplement: Supplementary file 2 — Supplementary material 2. [file 12672_2025_2905_MOESM2_ESM.png]

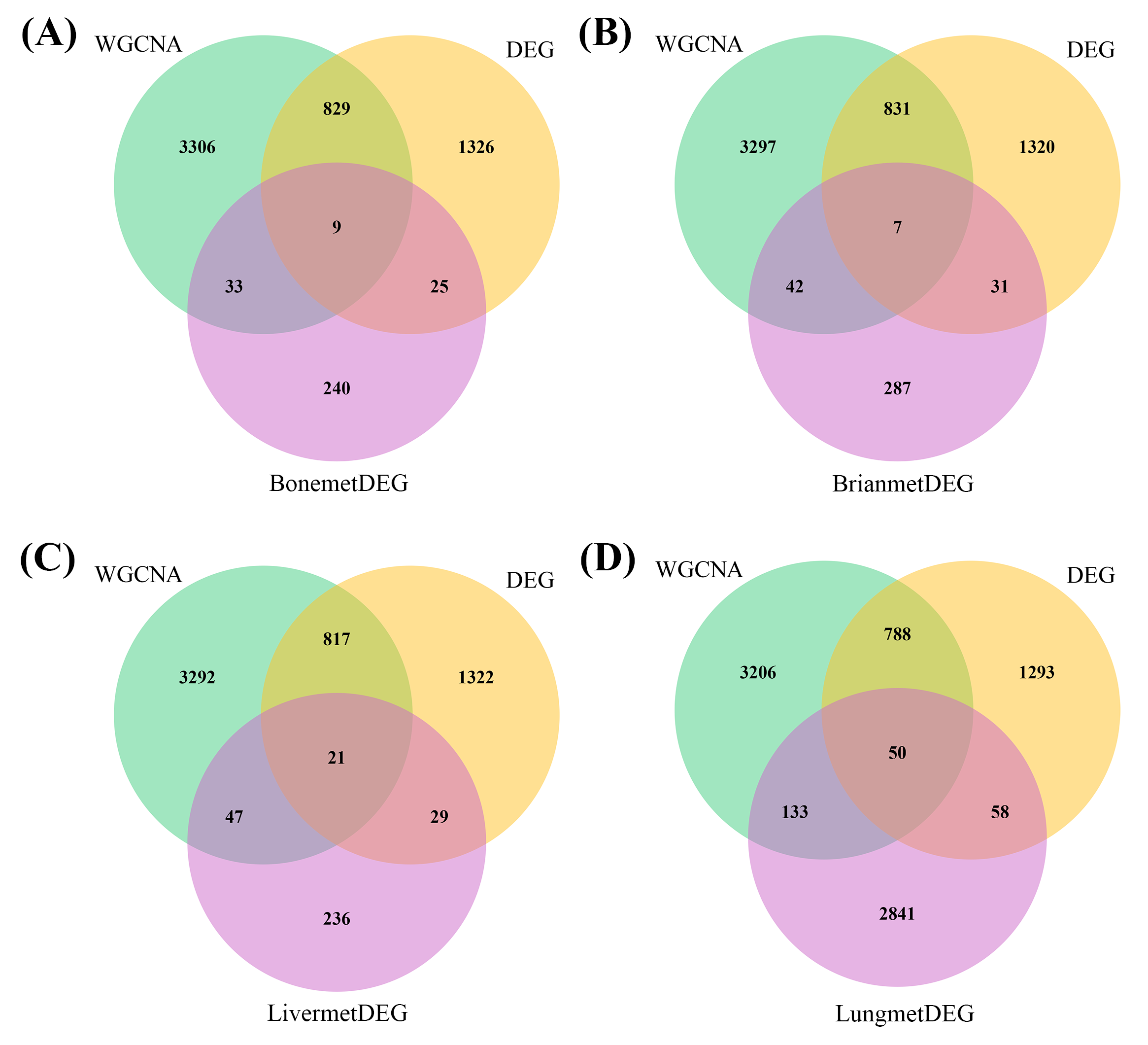

Supplement: Supplementary file 3 — Supplementary material 3. [file 12672_2025_2905_MOESM3_ESM.png]

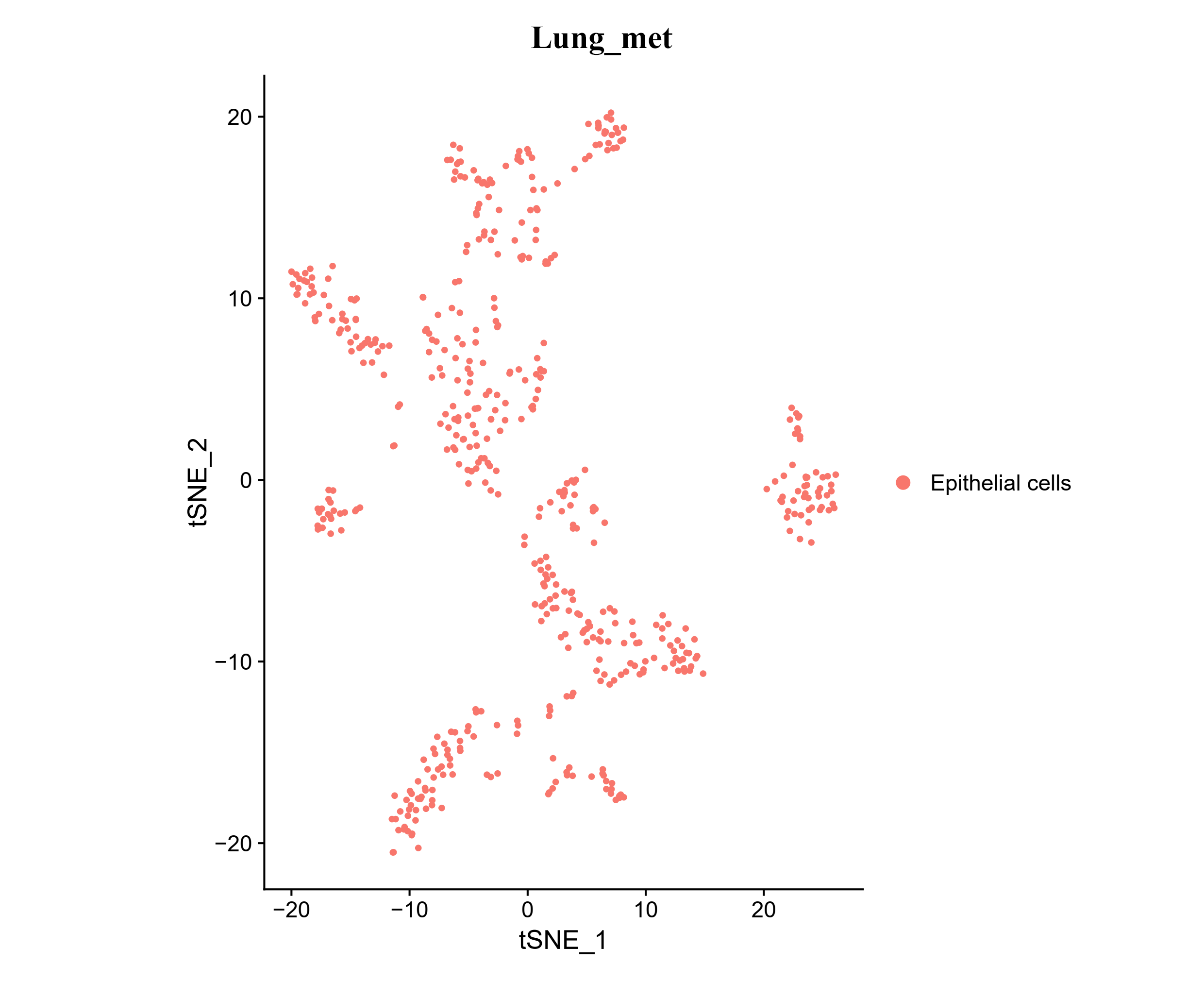

Supplement: Supplementary file 4 — Supplementary material 4. [file 12672_2025_2905_MOESM4_ESM.png]

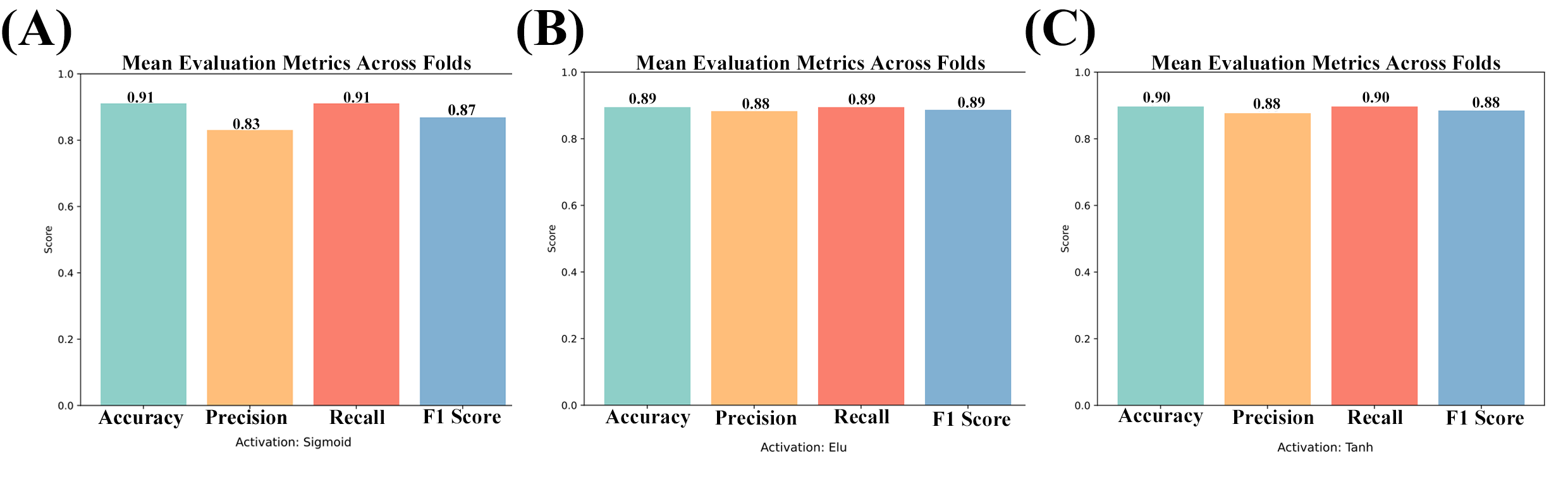

Supplement: Supplementary file 5 — Supplementary material 5. [file 12672_2025_2905_MOESM5_ESM.png]
